# Nonapotassium trialuminium hexa­phosphate

**DOI:** 10.1107/S160053681001305X

**Published:** 2010-04-17

**Authors:** Zuoliang Liu, Guochun Zhang, Jianxiu Zhang, Peizhen Fu, Yicheng Wu

**Affiliations:** a Key Laboratory of Functional Crystal and Laser Technology, Technical Institute of Physics and Chemistry, Chinese Academy of Sciences, Beijing 100190, People’s Republic of China

## Abstract

In the title compound, K_9_Al_3_(PO_4_)_6_, the anionic substructure is built of inter­linked [PO_4_] and [AlO_4_] tetra­hedra. Each O atom of the [AlO_4_] tetra­hedron is common to a positionally different [PO_4_] tetra­hedron; thus, each [AlO_4_] tetra­hedron is surrounded by four positionally different [PO_4_] tetra­hedra. On the other hand, each [PO_4_] tetra­hedron shares its two O atoms with two positionally different [AlO_4_] tetra­hedra; the other two phosphate O atoms are terminal ones coordinated by K atoms. The terminal O atoms are usually closer to the K atoms than the bridging O atoms between the [AlO_4_] and [PO_4_] tetra­hedra. There are nine symmetry-independent K atoms in the structure. The coordination numbers of the K atoms are 6 or 7 or 8 up to a distance of 3.31 Å. There are channels in the anionic substructure oriented along the [10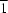] direction that are filled by K atoms.

## Related literature

For applications of metal phosphates, see: Barone & Nancollas (1978 ▶); Dickinson *et al.* (1996 ▶). For non-centrosymmetric phosphates with non-linear optical properties, see: Noor & Dam (1986 ▶); Aguilo & Wuensdregt (1985 ▶);  Masse & Grenier (1971 ▶). For the non-centrosymmetric structures of *A*_3_Al_2_(PO_4_)_3_ (*A* = K, Rb and Tl), which have three-dimensional [Al_2_P_3_O_12_]^3−^ frameworks, see: Nandini Devi & Vidyasagar (2000 ▶). For the structure of KAlP_2_O_7_, see: Ng & Calvo (1973 ▶);  

## Experimental

### 

#### Crystal data

K_9_Al_3_(PO_4_)_6_
*M* *_r_* = 1002.66
Monoclinic, 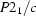

*a* = 20.289 (4) Å
*b* = 9.835 (2) Å
*c* = 13.521 (3) Å
β = 100.56 (3)°
*V* = 2652.2 (9) Å^3^
*Z* = 4
Mo *K*α radiation
μ = 2.02 mm^−1^
*T* = 113 K
0.26 × 0.20 × 0.18 mm

#### Data collection

Bruker SMART 1000 diffractometer
Absorption correction: numerical (*CrystalClear*; Rigaku/MSC, 2005 ▶) *T*_min_ = 0.622, *T*_max_ = 0.713
35263 measured reflections
11751 independent reflections
10169 reflections with *I* > 2σ(*I*)
*R*_int_ = 0.028

#### Refinement

*R*[*F* ^2^ > 2σ(*F* ^2^)] = 0.023
*wR*(*F* ^2^) = 0.059
*S* = 1.10
11751 reflections
380 parameters
Δρ_max_ = 0.54 e Å^−3^
Δρ_min_ = −0.52 e Å^−3^

### 

Data collection: *CrystalClear* (Rigaku/MSC, 2005 ▶); cell refinement: *CrystalClear*; data reduction: *CrystalClear*; program(s) used to solve structure: *SHELXS97* (Sheldrick, 2008 ▶); program(s) used to refine structure: *SHELXL97* (Sheldrick, 2008 ▶); molecular graphics: *Mercury* (Macrae *et al.*, 2006 ▶); software used to prepare material for publication: *CrystalStructure* (Rigaku/MSC, 2005 ▶).

## Supplementary Material

Crystal structure: contains datablocks I, global. DOI: 10.1107/S160053681001305X/fb2181sup1.cif

Structure factors: contains datablocks I. DOI: 10.1107/S160053681001305X/fb2181Isup2.hkl

Additional supplementary materials:  crystallographic information; 3D view; checkCIF report

## Figures and Tables

**Table 1:** Characterization of K—O coordination spheres; coordination number as well as minimal and maximal distances (Å) within the coordination spheres for each K atom are given

Atom K	Coordination number	K—O distances
K1	8	2.6202 (10)–3.2026 (12)
K2	7	2.5994 (9)–2.9721 (10)
K3	6	2.6337 (11)–2.9790 (11)
K4	6	2.7005 (10)–3.0451 (10)
K5	8	2.6787 (9)–3.3087 (12)
K6	7	2.6690 (10)–3.0404 (9)
K7	7	2.6369 (9)–3.0973 (10)
K8	7	2.5736 (9)–3.0747 (10)
K9	7	2.6965 (9)–3.3094 (11)

**Table 2:** Characterization of K—O coordination spheres; the minimal and maximal K—O distances (Å) for the terminal and bridging O atoms (there is only one bridging oxygen in the coordination spheres of K2, K3, and K8)

Atom K	K—O_terminal_ / K—O_bridge_ distances
K1	2.6202 (12)–3.2027 (18) / 3.0287 (14)–3.1684 (16)
K2	2.5994 (12)–2.9722 (14) / 2.8588 (14)
K3	2.6337 (13)–2.9790 (14) / 2.8348 (13)
K4	2.7006 (13)–2.7762 (15) / 2.9576 (15)–3.0451 (13)
K5	2.6787 (11)–3.3088 (19) / 3.1012 (15)–3.2476 (15)
K6	2.6690 (14)–2.9869 (16) / 2.8236 (13)–3.0405 (13)
K7	2.6368 (12)–2.9005 (15) / 2.7891 (14)–3.0974 (13)
K8	2.5737 (12)–3.0747 (13) / 2.9491 (13)
K9	2.6965 (2)–2.8865 (13) / 2.9783 (12)–3.3095 (16)

**Fig. 1.**
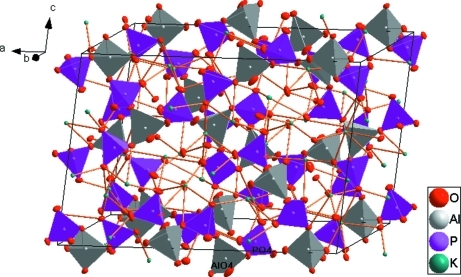
Unit cell of K9Al3(PO4)6. The displacement ellipsoids are drawn at the 90% probability level.

**Fig. 2.**
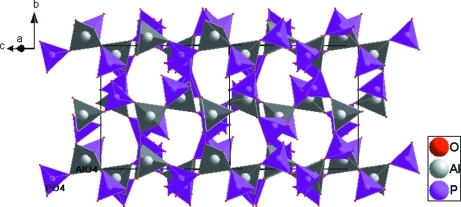
Anionic framework of K9Al3(PO4)6, viewed along [1 0 1].

**Fig. 3.**
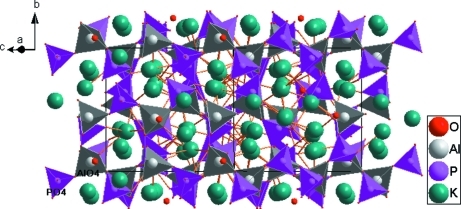
Anionic framework of K9Al3(PO4)6 filled with K atoms, viewed along [101].

## supplementary crystallographic information

### Comment

Various metal phosphates have been widely used due to their good optical and chemical properties. For example, Ca_5_(PO_4_)_3_F has been used in dentistry (Barone & Nancollas, 1978) and Sr_5_(PO_4_)_3_F (Dickinson *et al.*, 1996) has been used as a laser crystal in laser technology. Especially, some non-centrosymmetric phosphates have been used as important crystals with nonlinear optical properties, such as KH_2_PO_4_ (KDP) (Noor & Dam, 1986), NH_4_H_2_PO_4_ (ADP) (Aguilo & Wuensdregt, 1985) and KTiOPO_4_ (KTP) (Masse & Grenier, 1971). Aluminophosphates have attracted much attention because of their diverse structures.

Aluminophosphates contain 1D, 2D or 3D infinite frameworks with varying chemical composition. Nandini Devi & Vidyasagar (2000) reported non-centrosymmetric structures of A_3_Al_2_(PO_4_)_3_ (A=K, Rb and Tl). These compounds have 3D [Al_2_P_3_O_12_]^3-^ frameworks. The latter study has inspired us to investigate the A_2_O—Al_2_O_3_—P_2_O_5_ (A=K, Rb, Cs) system in order to search for new functional materials. As a result of our study a new aluminophosphate, the title structure K_9_Al_3_(PO_4_)_6_, has been discovered.

In K_9_Al_3_(PO_4_)_6_, all the aluminium and phosphorus atoms adopt the tetrahedral coordination. Each [AlO_4_] tetrahedron shares each of its O atoms with a positionally different neighbour [PO_4_] tetrahedron, while each [PO_4_] tetrahedron shares its two O atoms with two different neighbour [AlO_4_] tetrahedral. There are two pairs of chemically different O atoms around the P atoms: The terminal and the bridging oxygens that are involved in P-O-Al connections (Fig. 1). The P-O distance to the bridging oxygens vary in the interval 1.5645 (10) - 1.5881 (8) Å, while the P-O distances to the terminal oxygens are in the interval 1.4993 (10) - 1.5087 (9) Å.

There are channels in the anionic substructure along [1 0 1] (Fig. 2). These channels are filled by K atoms (Fig. 3). The coordination numbers of K atoms are 6 or 7 or 8 up to the distance 3.31 Å (Tab. 1). The terminal phosphate oxygens tend to be closer to K atoms than the bridging ones (Tab. 2).

### Experimental

Single crystals of K_9_Al_3_(PO_4_)_6_ have been obtained by the high temperature solution method in a electric resistance furnace. Starting materials of the analytical grade KH_2_PO_4_ (136.15 g) and K_2_CO_3_ (69.03 g), high purity Al_2_O_3_ (51.08 g) and KF (58.33 g), in the respective molar ratio 2:1:1:2, were mixed and melt in a platinum crucible with a diameter of 60 mm and a height of 60 mm at 1273 K. The solution was stirred with a platinum plate for 24 hours. After the solution had been cooled to 1123 K at a rate of 10 Kh^-1^, a platinum wire attached to an alumina shaft was slowly dipped into the solution, which was then followed by a slow cooling at the rate of 0.5 Kh^-1^. Thus, a few colourless, transparent plate K_9_Al_3_(PO_4_)_6_ crystals with typical size of 3 × 3 × 0.5 mm crystallized on the platinum wire. After one week, the crystals were drawn out from the solution at 1050 K and cooled down to room temperature at the rate of 10 Kh^-1^.

### Refinement

All the atomic have been refined anisotropically. The maximal (0.542 eÅ^-3^) and minimal (-0.519 eÅ^-3^) electron density peaks are situated 0.67 Å from O16 and 0.56 Å from P4, respectively.

### Crystal data

**Table:**

K_9_Al_3_(PO_4_)_6_	*F*(000) = 1968
*M**_r_* = 1002.66	*D*_x_ = 2.511 Mg m^−^^3^
Monoclinic, *P*2_1_/*c*	Mo *K*α radiation, λ = 0.71073 Å
Hall symbol: -P 2ybc	Cell parameters from 11243 reflections
*a* = 20.289 (4) Å	θ = 1.5–36.1°
*b* = 9.835 (2) Å	µ = 2.02 mm^−^^1^
*c* = 13.521 (3) Å	*T* = 113 K
β = 100.56 (3)°	Block, colorless
*V* = 2652.2 (9) Å^3^	0.26 × 0.20 × 0.18 mm
*Z* = 4	

### Data collection

**Table:**

Bruker SMART 1000 diffractometer	11751 independent reflections
Radiation source: rotating anode	10169 reflections with *I* > 2σ(*I*)
confocal	*R*_int_ = 0.028
Detector resolution: 7.31 pixels mm^-1^	θ_max_ = 36.4°, θ_min_ = 2.0°
ω and φ scans	*h* = −32→32
Absorption correction: numerical (*CrystalClear*; Rigaku/MSC, 2005)	*k* = −15→14
*T*_min_ = 0.622, *T*_max_ = 0.713	*l* = −21→21
35263 measured reflections	

### Refinement

**Table:**

Refinement on *F*^2^	Primary atom site location: structure-invariant direct methods
Least-squares matrix: full	Secondary atom site location: difference Fourier map
*R*[*F*^2^ > 2σ(*F*^2^)] = 0.023	*w* = 1/[σ^2^(*F*_o_^2^) + (0.0289*P*)^2^ + 0.2004*P*] where *P* = (*F*_o_^2^ + 2*F*_c_^2^)/3
*wR*(*F*^2^) = 0.059	(Δ/σ)_max_ = 0.002
*S* = 1.10	Δρ_max_ = 0.54 e Å^−^^3^
11751 reflections	Δρ_min_ = −0.52 e Å^−^^3^
380 parameters	Extinction correction: *SHELXL97* (Sheldrick, 2008), Fc^*^=kFc[1+0.001xFc^2^λ^3^/sin(2θ)]^-1/4^
0 restraints	Extinction coefficient: 0.0094 (3)
0 constraints	

### Special details

**Table:**

Geometry. All esds (except the esd in the dihedral angle between two l.s. planes) are estimated using the full covariance matrix. The cell esds are taken into account individually in the estimation of esds in distances, angles and torsion angles; correlations between esds in cell parameters are only used when they are defined by crystal symmetry. An approximate (isotropic) treatment of cell esds is used for estimating esds involving l.s. planes.
Refinement. Refinement of *F*^2^ against ALL reflections. The weighted *R*-factor wR and goodness of fit *S* are based on *F*^2^, conventional *R*-factors *R* are based on *F*, with *F* set to zero for negative *F*^2^. The threshold expression of *F*^2^ > σ(*F*^2^) is used only for calculating *R*-factors(gt) etc. and is not relevant to the choice of reflections for refinement. *R*-factors based on *F*^2^ are statistically about twice as large as those based on *F*, and *R*- factors based on ALL data will be even larger.

### Fractional atomic coordinates and isotropic or equivalent isotropic displacement parameters (Å^2^)

**Table:**

	*x*	*y*	*z*	*U*_iso_*/*U*_eq_	
P1	0.298252 (13)	0.34947 (3)	0.33480 (2)	0.00581 (5)	
P2	0.533853 (13)	0.58644 (3)	0.36009 (2)	0.00594 (5)	
P3	0.187195 (13)	0.57954 (3)	0.69175 (2)	0.00569 (5)	
P4	−0.043493 (13)	0.14690 (3)	0.10689 (2)	0.00566 (5)	
P5	0.356473 (13)	0.65268 (3)	0.99111 (2)	0.00556 (5)	
P6	0.134551 (13)	0.14154 (3)	0.50989 (2)	0.00546 (5)	
Al1	0.089399 (16)	0.07540 (3)	0.02839 (2)	0.00532 (6)	
Al2	0.248584 (16)	0.06371 (3)	0.38928 (2)	0.00494 (6)	
Al3	0.404521 (16)	0.56335 (3)	0.44487 (2)	0.00506 (6)	
O1	0.30136 (4)	0.20134 (8)	0.37989 (6)	0.01040 (14)	
O2	0.30827 (5)	0.34641 (8)	0.22768 (6)	0.01385 (16)	
O3	0.23519 (4)	0.41939 (8)	0.35084 (7)	0.01469 (16)	
O4	0.36066 (5)	0.41798 (8)	0.40128 (7)	0.01553 (18)	
O5	0.57090 (4)	0.71131 (8)	0.40329 (6)	0.01132 (15)	
O6	0.54290 (4)	0.54825 (8)	0.25534 (6)	0.00961 (14)	
O7	0.45662 (4)	0.60301 (9)	0.35924 (6)	0.01063 (14)	
O8	0.55470 (4)	0.45990 (7)	0.43131 (6)	0.00885 (14)	
O9	0.24384 (4)	0.56940 (8)	0.63432 (6)	0.01142 (15)	
O10	0.11777 (4)	0.54245 (7)	0.62321 (6)	0.00823 (14)	
O11	0.17814 (4)	0.71622 (8)	0.73771 (6)	0.01017 (14)	
O12	0.19385 (4)	0.46544 (8)	0.77559 (6)	0.00888 (14)	
O13	−0.09231 (4)	0.25724 (7)	0.06586 (6)	0.00840 (13)	
O14	0.01158 (4)	0.14074 (7)	0.03954 (6)	0.00818 (14)	
O15	−0.07987 (4)	0.00350 (7)	0.09080 (6)	0.00757 (13)	
O16	−0.01117 (4)	0.16378 (8)	0.21565 (6)	0.01014 (14)	
O17	0.34118 (4)	0.65873 (8)	1.09580 (6)	0.01213 (15)	
O18	0.35063 (4)	0.79852 (7)	0.94185 (6)	0.00881 (14)	
O19	0.42285 (4)	0.58847 (8)	0.98471 (7)	0.01214 (15)	
O20	0.29847 (4)	0.57812 (8)	0.91867 (6)	0.01111 (15)	
O21	0.20437 (4)	0.09364 (8)	0.48639 (6)	0.00835 (13)	
O22	0.11949 (4)	0.05905 (7)	0.59700 (6)	0.00888 (14)	
O23	0.14854 (4)	0.29353 (7)	0.54464 (6)	0.00891 (14)	
O24	0.08212 (4)	0.14116 (8)	0.41564 (6)	0.01017 (14)	
K1	0.287854 (12)	0.17131 (2)	0.079146 (18)	0.00949 (4)	
K2	0.420108 (12)	0.44811 (2)	0.178588 (19)	0.01038 (4)	
K3	0.233669 (12)	0.57239 (2)	0.160286 (19)	0.01005 (4)	
K4	0.551481 (12)	0.66477 (2)	0.075535 (18)	0.00984 (4)	
K5	0.370503 (13)	0.84243 (2)	0.243608 (19)	0.01203 (5)	
K6	0.054431 (12)	0.67635 (2)	0.106782 (18)	0.00886 (4)	
K7	0.213027 (11)	0.66267 (2)	0.427477 (18)	0.00950 (4)	
K8	0.058558 (12)	0.93957 (2)	0.292875 (18)	0.01007 (4)	
K9	0.116931 (12)	0.29859 (2)	0.266699 (19)	0.01164 (5)	

### Atomic displacement parameters (Å^2^)

**Table:**

	*U*^11^	*U*^22^	*U*^33^	*U*^12^	*U*^13^	*U*^23^
P1	0.00581 (11)	0.00550 (10)	0.00599 (12)	−0.00069 (8)	0.00070 (9)	−0.00048 (8)
P2	0.00578 (11)	0.00678 (10)	0.00535 (11)	0.00080 (8)	0.00123 (9)	0.00050 (8)
P3	0.00625 (11)	0.00554 (10)	0.00511 (11)	−0.00021 (8)	0.00058 (9)	0.00017 (8)
P4	0.00518 (11)	0.00625 (10)	0.00552 (11)	−0.00004 (8)	0.00088 (8)	−0.00072 (8)
P5	0.00535 (11)	0.00497 (10)	0.00632 (12)	−0.00068 (8)	0.00096 (9)	−0.00019 (8)
P6	0.00536 (11)	0.00549 (10)	0.00549 (11)	0.00029 (8)	0.00088 (8)	0.00015 (8)
Al1	0.00504 (13)	0.00500 (12)	0.00582 (14)	−0.00004 (9)	0.00069 (11)	−0.00028 (10)
Al2	0.00504 (13)	0.00437 (12)	0.00522 (14)	0.00060 (9)	0.00042 (10)	−0.00012 (10)
Al3	0.00478 (13)	0.00426 (12)	0.00582 (14)	0.00029 (9)	0.00011 (10)	−0.00015 (10)
O1	0.0108 (3)	0.0065 (3)	0.0133 (4)	−0.0026 (2)	0.0007 (3)	0.0015 (3)
O2	0.0213 (4)	0.0131 (4)	0.0086 (4)	0.0002 (3)	0.0065 (3)	0.0005 (3)
O3	0.0110 (4)	0.0129 (4)	0.0217 (5)	0.0031 (3)	0.0072 (3)	−0.0006 (3)
O4	0.0157 (4)	0.0068 (3)	0.0195 (4)	−0.0029 (3)	−0.0091 (3)	0.0002 (3)
O5	0.0147 (4)	0.0073 (3)	0.0115 (4)	−0.0024 (3)	0.0013 (3)	0.0000 (3)
O6	0.0100 (3)	0.0133 (3)	0.0060 (3)	0.0015 (3)	0.0026 (3)	−0.0003 (3)
O7	0.0071 (3)	0.0172 (4)	0.0079 (4)	0.0033 (3)	0.0023 (3)	0.0021 (3)
O8	0.0114 (3)	0.0071 (3)	0.0071 (3)	0.0011 (2)	−0.0006 (3)	0.0011 (3)
O9	0.0096 (3)	0.0145 (4)	0.0112 (4)	−0.0004 (3)	0.0045 (3)	−0.0001 (3)
O10	0.0077 (3)	0.0085 (3)	0.0073 (3)	0.0005 (2)	−0.0018 (3)	−0.0011 (3)
O11	0.0130 (4)	0.0069 (3)	0.0100 (4)	0.0001 (3)	0.0004 (3)	−0.0022 (3)
O12	0.0094 (3)	0.0087 (3)	0.0073 (3)	−0.0019 (2)	−0.0016 (3)	0.0030 (3)
O13	0.0081 (3)	0.0079 (3)	0.0094 (3)	0.0018 (2)	0.0020 (3)	0.0008 (3)
O14	0.0064 (3)	0.0083 (3)	0.0108 (4)	0.0005 (2)	0.0042 (3)	0.0004 (3)
O15	0.0091 (3)	0.0068 (3)	0.0072 (3)	−0.0023 (2)	0.0026 (3)	−0.0008 (2)
O16	0.0112 (4)	0.0116 (3)	0.0069 (4)	0.0002 (3)	−0.0004 (3)	−0.0022 (3)
O17	0.0165 (4)	0.0117 (3)	0.0093 (4)	−0.0007 (3)	0.0054 (3)	0.0000 (3)
O18	0.0083 (3)	0.0057 (3)	0.0119 (4)	−0.0020 (2)	0.0005 (3)	0.0018 (3)
O19	0.0080 (3)	0.0113 (3)	0.0175 (4)	0.0025 (3)	0.0033 (3)	0.0004 (3)
O20	0.0107 (3)	0.0078 (3)	0.0130 (4)	−0.0045 (3)	−0.0027 (3)	0.0008 (3)
O21	0.0077 (3)	0.0107 (3)	0.0070 (3)	0.0023 (2)	0.0022 (3)	0.0001 (3)
O22	0.0101 (3)	0.0092 (3)	0.0078 (3)	−0.0004 (2)	0.0028 (3)	0.0015 (3)
O23	0.0072 (3)	0.0051 (3)	0.0139 (4)	0.0009 (2)	0.0006 (3)	−0.0007 (3)
O24	0.0083 (3)	0.0133 (3)	0.0079 (4)	0.0006 (3)	−0.0011 (3)	−0.0007 (3)
K1	0.00909 (9)	0.00962 (9)	0.00989 (10)	−0.00004 (7)	0.00210 (8)	0.00004 (7)
K2	0.01006 (9)	0.00876 (9)	0.01141 (11)	−0.00028 (7)	−0.00044 (8)	−0.00135 (8)
K3	0.00859 (9)	0.00980 (9)	0.01165 (11)	−0.00057 (7)	0.00156 (8)	−0.00163 (8)
K4	0.01018 (9)	0.01036 (9)	0.00913 (10)	−0.00115 (7)	0.00219 (8)	0.00072 (7)
K5	0.01415 (11)	0.01086 (10)	0.01155 (11)	−0.00281 (7)	0.00362 (8)	−0.00148 (8)
K6	0.00872 (9)	0.00997 (9)	0.00819 (10)	0.00093 (7)	0.00236 (7)	0.00127 (7)
K7	0.00723 (9)	0.01039 (9)	0.01103 (10)	0.00059 (7)	0.00207 (8)	0.00037 (7)
K8	0.01277 (10)	0.00765 (9)	0.01026 (10)	0.00019 (7)	0.00335 (8)	0.00097 (7)
K9	0.01126 (10)	0.01316 (10)	0.01026 (10)	−0.00163 (7)	0.00134 (8)	0.00316 (8)

### Geometric parameters (Å, °)

**Table:**

P1—O2	1.4993 (10)	O16—K6^ix^	2.7060 (11)
P1—O3	1.5030 (9)	O16—K8^xi^	2.7225 (10)
P1—O4	1.5645 (10)	O16—K8^ix^	2.8730 (10)
P1—O1	1.5760 (8)	O16—K9	2.8864 (10)
P2—O5	1.5011 (9)	O17—K3^xii^	2.6337 (11)
P2—O6	1.5087 (9)	O17—K5^xii^	2.6788 (10)
P2—O7	1.5734 (9)	O17—K2^xii^	2.7296 (10)
P2—O8	1.5829 (8)	O18—Al3^viii^	1.7396 (8)
P3—O9	1.5036 (10)	O18—K7^viii^	2.7891 (10)
P3—O11	1.5062 (8)	O18—K5^viii^	3.1088 (10)
P3—O10	1.5803 (9)	O19—K4^iv^	2.7005 (10)
P3—O12	1.5831 (8)	O19—K4^xii^	2.7761 (11)
P4—O13	1.5049 (8)	O19—K2^xii^	2.9721 (10)
P4—O16	1.5056 (9)	O19—K5^viii^	3.3087 (12)
P4—O14	1.5667 (9)	O20—Al2^vi^	1.7264 (8)
P4—O15	1.5881 (8)	O20—K7^viii^	3.0973 (10)
P5—O19	1.5043 (9)	O20—K5^viii^	3.1013 (12)
P5—O17	1.5049 (9)	O21—K3^vi^	2.8347 (9)
P5—O20	1.5682 (9)	O21—K1^vi^	3.0012 (10)
P5—O18	1.5767 (8)	O22—K3^vi^	2.6539 (10)
P6—O24	1.5029 (10)	O22—K6^vi^	2.6802 (9)
P6—O22	1.5068 (9)	O22—K9^vi^	2.6965 (9)
P6—O23	1.5770 (8)	O23—Al1^vi^	1.7472 (8)
P6—O21	1.5794 (9)	O23—K1^vi^	2.8007 (10)
Al1—O14	1.7366 (9)	O23—K9^vi^	3.3094 (11)
Al1—O10^i^	1.7457 (9)	O24—K8^xi^	2.5736 (9)
Al1—O23^i^	1.7472 (8)	O24—K9	2.7337 (10)
Al1—O15^ii^	1.7671 (9)	O24—K6^ix^	2.7531 (10)
Al2—O20^i^	1.7264 (8)	K1—O9^i^	2.6837 (10)
Al2—O1	1.7449 (9)	K1—O23^i^	2.8007 (10)
Al2—O21	1.7457 (10)	K1—O5^v^	2.8591 (11)
Al2—O12^i^	1.7472 (10)	K1—O21^i^	3.0012 (10)
Al3—O4	1.7295 (9)	K1—O1^i^	3.0286 (10)
Al3—O18^iii^	1.7396 (8)	K1—O4^i^	3.1684 (13)
Al3—O8^iv^	1.7411 (10)	K1—O3^i^	3.2026 (12)
Al3—O7	1.7490 (10)	K2—O5^v^	2.5994 (9)
O1—K4^v^	2.9576 (11)	K2—O17^xiii^	2.7296 (10)
O1—K1^vi^	3.0286 (10)	K2—O19^xiii^	2.9721 (10)
O2—K1	2.6202 (10)	K3—O17^xiii^	2.6337 (11)
O2—K2	2.6725 (11)	K3—O22^i^	2.6539 (10)
O2—K3	2.7486 (10)	K3—O11^iii^	2.6683 (9)
O3—K7	2.6779 (10)	K3—O21^i^	2.8347 (9)
O3—K9	2.7316 (11)	K4—O19^iv^	2.7005 (10)
O3—K3	2.9790 (11)	K4—O5^iii^	2.7205 (10)
O3—K1^vi^	3.2026 (12)	K4—O19^xiii^	2.7761 (11)
O4—K4^v^	3.0451 (10)	K4—O1^vii^	2.9576 (11)
O4—K1^vi^	3.1684 (13)	K4—O4^vii^	3.0451 (10)
O5—K2^vii^	2.5994 (9)	K5—O6^vii^	2.6787 (9)
O5—K4^viii^	2.7205 (10)	K5—O17^xiii^	2.6788 (10)
O5—K1^vii^	2.8591 (11)	K5—O9^iii^	2.8544 (12)
O6—K5^v^	2.6787 (9)	K5—O20^iii^	3.1013 (12)
O6—K2	2.7029 (11)	K5—O18^iii^	3.1088 (10)
O6—K4	2.7219 (9)	K5—O8^vii^	3.2476 (11)
O7—K2	2.8587 (10)	K5—O19^iii^	3.3087 (12)
O7—K5	3.1685 (10)	K6—O13^x^	2.6690 (10)
O8—Al3^iv^	1.7411 (10)	K6—O22^i^	2.6803 (9)
O8—K5^v^	3.2476 (11)	K6—O16^xiv^	2.7060 (11)
O9—K1^vi^	2.6837 (10)	K6—O24^xiv^	2.7531 (10)
O9—K5^viii^	2.8544 (12)	K6—O14^x^	2.8235 (10)
O9—K7	2.9005 (11)	K6—O11^iii^	2.9867 (12)
O10—Al1^vi^	1.7457 (9)	K6—O10^iii^	3.0404 (9)
O10—K8^viii^	2.7834 (11)	K7—O13^xiv^	2.6369 (9)
O10—K6^viii^	3.0404 (9)	K7—O18^iii^	2.7891 (10)
O11—K3^viii^	2.6683 (9)	K7—O11^iii^	2.7995 (10)
O11—K7^viii^	2.7995 (10)	K7—O15^xiv^	3.0928 (10)
O11—K6^viii^	2.9867 (12)	K7—O20^iii^	3.0973 (10)
O11—K8^viii^	3.0747 (10)	K8—O24^xv^	2.5736 (9)
O12—Al2^vi^	1.7472 (10)	K8—O13^xiv^	2.6179 (9)
O12—K8^viii^	2.9492 (10)	K8—O16^xv^	2.7225 (10)
O12—K9^vi^	3.0206 (9)	K8—O10^iii^	2.7834 (11)
O13—K8^ix^	2.6179 (9)	K8—O16^xiv^	2.8730 (10)
O13—K7^ix^	2.6369 (9)	K8—O12^iii^	2.9492 (10)
O13—K6^x^	2.6690 (10)	K8—O11^iii^	3.0747 (10)
O14—K6^x^	2.8235 (10)	K9—O22^i^	2.6965 (9)
O15—Al1^ii^	1.7671 (9)	K9—O15^xiv^	2.9782 (9)
O15—K9^ix^	2.9782 (9)	K9—O12^i^	3.0206 (9)
O15—K7^ix^	3.0928 (10)	K9—O23^i^	3.3094 (11)
			
O2—P1—O3	114.74 (6)	O6—K4—O1^vii^	95.55 (4)
O2—P1—O4	108.96 (6)	O19^xiii^—K4—O1^vii^	162.75 (3)
O3—P1—O4	109.93 (5)	O19^iv^—K4—O4^vii^	130.06 (3)
O2—P1—O1	110.62 (5)	O5^iii^—K4—O4^vii^	63.12 (3)
O3—P1—O1	109.99 (5)	O6—K4—O4^vii^	112.61 (3)
O4—P1—O1	101.83 (5)	O19^xiii^—K4—O4^vii^	138.74 (2)
O5—P2—O6	115.50 (5)	O1^vii^—K4—O4^vii^	47.89 (2)
O5—P2—O7	110.19 (5)	O6^vii^—K5—O17^xiii^	124.71 (3)
O6—P2—O7	108.14 (5)	O6^vii^—K5—O9^iii^	107.49 (3)
O5—P2—O8	110.34 (5)	O17^xiii^—K5—O9^iii^	76.28 (3)
O6—P2—O8	108.08 (5)	O6^vii^—K5—O20^iii^	101.89 (3)
O7—P2—O8	103.92 (5)	O17^xiii^—K5—O20^iii^	131.91 (3)
O9—P3—O11	115.78 (5)	O9^iii^—K5—O20^iii^	79.22 (3)
O9—P3—O10	111.44 (5)	O6^vii^—K5—O18^iii^	121.40 (3)
O11—P3—O10	106.55 (4)	O17^xiii^—K5—O18^iii^	107.24 (3)
O9—P3—O12	110.48 (5)	O9^iii^—K5—O18^iii^	109.57 (3)
O11—P3—O12	109.75 (5)	O20^iii^—K5—O18^iii^	45.61 (2)
O10—P3—O12	101.90 (5)	O6^vii^—K5—O7	104.90 (3)
O13—P4—O16	114.83 (5)	O17^xiii^—K5—O7	83.91 (3)
O13—P4—O14	107.75 (5)	O9^iii^—K5—O7	147.55 (2)
O16—P4—O14	110.03 (5)	O20^iii^—K5—O7	96.03 (2)
O13—P4—O15	109.38 (5)	O18^iii^—K5—O7	52.43 (2)
O16—P4—O15	109.88 (5)	O6^vii^—K5—O8^vii^	48.81 (2)
O14—P4—O15	104.43 (4)	O17^xiii^—K5—O8^vii^	76.48 (3)
O19—P5—O17	114.32 (6)	O9^iii^—K5—O8^vii^	90.66 (3)
O19—P5—O20	110.19 (5)	O20^iii^—K5—O8^vii^	144.56 (2)
O17—P5—O20	110.20 (5)	O18^iii^—K5—O8^vii^	159.77 (2)
O19—P5—O18	110.88 (5)	O7—K5—O8^vii^	109.55 (3)
O17—P5—O18	110.42 (5)	O6^vii^—K5—O19^iii^	75.39 (3)
O20—P5—O18	99.89 (5)	O17^xiii^—K5—O19^iii^	149.41 (2)
O24—P6—O22	116.61 (5)	O9^iii^—K5—O19^iii^	122.93 (3)
O24—P6—O23	108.41 (4)	O20^iii^—K5—O19^iii^	46.16 (2)
O22—P6—O23	109.19 (5)	O18^iii^—K5—O19^iii^	46.45 (2)
O24—P6—O21	110.50 (5)	O7—K5—O19^iii^	67.55 (3)
O22—P6—O21	108.33 (5)	O8^vii^—K5—O19^iii^	122.41 (2)
O23—P6—O21	102.92 (4)	O13^x^—K6—O22^i^	86.84 (3)
O14—Al1—O10^i^	111.38 (5)	O13^x^—K6—O16^xiv^	168.34 (2)
O14—Al1—O23^i^	109.29 (4)	O22^i^—K6—O16^xiv^	104.31 (3)
O10^i^—Al1—O23^i^	105.73 (4)	O13^x^—K6—O24^xiv^	112.32 (4)
O14—Al1—O15^ii^	107.00 (5)	O22^i^—K6—O24^xiv^	112.38 (3)
O10^i^—Al1—O15^ii^	110.15 (4)	O16^xiv^—K6—O24^xiv^	66.94 (4)
O23^i^—Al1—O15^ii^	113.35 (5)	O13^x^—K6—O14^x^	53.63 (3)
O20^i^—Al2—O1	107.57 (5)	O22^i^—K6—O14^x^	133.66 (3)
O20^i^—Al2—O21	108.87 (4)	O16^xiv^—K6—O14^x^	117.48 (3)
O1—Al2—O21	109.31 (4)	O24^xiv^—K6—O14^x^	70.39 (3)
O20^i^—Al2—O12^i^	108.74 (4)	O13^x^—K6—O11^iii^	94.96 (3)
O1—Al2—O12^i^	111.28 (4)	O22^i^—K6—O11^iii^	88.10 (3)
O21—Al2—O12^i^	110.98 (4)	O16^xiv^—K6—O11^iii^	82.20 (3)
O4—Al3—O18^iii^	110.83 (5)	O24^xiv^—K6—O11^iii^	146.01 (3)
O4—Al3—O8^iv^	110.07 (4)	O14^x^—K6—O11^iii^	115.41 (3)
O18^iii^—Al3—O8^iv^	108.12 (4)	O13^x^—K6—O10^iii^	69.86 (2)
O4—Al3—O7	107.13 (5)	O22^i^—K6—O10^iii^	125.73 (3)
O18^iii^—Al3—O7	105.30 (4)	O16^xiv^—K6—O10^iii^	100.27 (3)
O8^iv^—Al3—O7	115.31 (4)	O24^xiv^—K6—O10^iii^	121.72 (2)
O2—K1—O9^i^	112.37 (3)	O14^x^—K6—O10^iii^	67.14 (3)
O2—K1—O23^i^	93.41 (4)	O11^iii^—K6—O10^iii^	48.47 (2)
O9^i^—K1—O23^i^	77.18 (3)	O13^xiv^—K7—O3	123.45 (3)
O2—K1—O5^v^	80.25 (4)	O13^xiv^—K7—O18^iii^	150.88 (2)
O9^i^—K1—O5^v^	118.54 (3)	O3—K7—O18^iii^	84.97 (3)
O23^i^—K1—O5^v^	164.26 (2)	O13^xiv^—K7—O11^iii^	78.51 (4)
O2—K1—O21^i^	79.16 (3)	O3—K7—O11^iii^	93.31 (3)
O9^i^—K1—O21^i^	127.19 (3)	O18^iii^—K7—O11^iii^	95.01 (4)
O23^i^—K1—O21^i^	50.22 (2)	O13^xiv^—K7—O9	96.66 (4)
O5^v^—K1—O21^i^	114.15 (3)	O3—K7—O9	93.94 (3)
O2—K1—O1^i^	112.50 (3)	O18^iii^—K7—O9	86.56 (4)
O9^i^—K1—O1^i^	134.51 (3)	O11^iii^—K7—O9	172.69 (2)
O23^i^—K1—O1^i^	93.09 (4)	O13^xiv^—K7—O15^xiv^	51.54 (2)
O5^v^—K1—O1^i^	76.36 (4)	O3—K7—O15^xiv^	73.92 (3)
O21^i^—K1—O1^i^	56.35 (3)	O18^iii^—K7—O15^xiv^	157.43 (2)
O2—K1—O4^i^	137.20 (3)	O11^iii^—K7—O15^xiv^	94.11 (3)
O9^i^—K1—O4^i^	101.05 (3)	O9—K7—O15^xiv^	87.00 (3)
O23^i^—K1—O4^i^	120.30 (3)	O13^xiv^—K7—O20^iii^	103.94 (3)
O5^v^—K1—O4^i^	60.10 (3)	O3—K7—O20^iii^	125.85 (3)
O21^i^—K1—O4^i^	101.67 (3)	O18^iii^—K7—O20^iii^	47.93 (2)
O1^i^—K1—O4^i^	46.26 (2)	O11^iii^—K7—O20^iii^	70.36 (3)
O2—K1—O3^i^	154.13 (3)	O9—K7—O20^iii^	105.91 (3)
O9^i^—K1—O3^i^	87.31 (3)	O15^xiv^—K7—O20^iii^	154.20 (2)
O23^i^—K1—O3^i^	74.07 (4)	O24^xv^—K8—O13^xiv^	93.69 (3)
O5^v^—K1—O3^i^	105.70 (4)	O24^xv^—K8—O16^xv^	69.23 (3)
O21^i^—K1—O3^i^	75.45 (3)	O13^xiv^—K8—O16^xv^	151.96 (3)
O1^i^—K1—O3^i^	47.66 (2)	O24^xv^—K8—O10^iii^	115.18 (3)
O4^i^—K1—O3^i^	46.44 (3)	O13^xiv^—K8—O10^iii^	123.38 (3)
O5^v^—K2—O2	84.23 (3)	O16^xv^—K8—O10^iii^	84.54 (3)
O5^v^—K2—O6	110.99 (3)	O24^xv^—K8—O16^xiv^	140.75 (3)
O2—K2—O6	143.69 (3)	O13^xiv^—K8—O16^xiv^	54.79 (3)
O5^v^—K2—O17^xiii^	125.94 (3)	O16^xv^—K8—O16^xiv^	127.063 (19)
O2—K2—O17^xiii^	85.69 (3)	O10^iii^—K8—O16^xiv^	102.63 (2)
O6—K2—O17^xiii^	108.13 (3)	O24^xv^—K8—O12^iii^	75.02 (3)
O5^v^—K2—O7	144.48 (3)	O13^xiv^—K8—O12^iii^	98.75 (3)
O2—K2—O7	94.94 (3)	O16^xv^—K8—O12^iii^	97.98 (3)
O6—K2—O7	53.24 (3)	O10^iii^—K8—O12^iii^	50.65 (3)
O17^xiii^—K2—O7	89.24 (3)	O16^xiv^—K8—O12^iii^	127.26 (2)
O5^v^—K2—O19^xiii^	91.51 (3)	O24^xv^—K8—O11^iii^	118.61 (3)
O2—K2—O19^xiii^	123.16 (3)	O13^xiv^—K8—O11^iii^	73.93 (3)
O6—K2—O19^xiii^	90.25 (3)	O16^xv^—K8—O11^iii^	133.49 (3)
O17^xiii^—K2—O19^xiii^	52.44 (3)	O10^iii^—K8—O11^iii^	49.66 (3)
O7—K2—O19^xiii^	117.53 (3)	O16^xiv^—K8—O11^iii^	78.05 (3)
O17^xiii^—K3—O22^i^	141.01 (3)	O12^iii^—K8—O11^iii^	49.56 (2)
O17^xiii^—K3—O11^iii^	108.28 (3)	O22^i^—K9—O3	88.63 (4)
O22^i^—K3—O11^iii^	95.71 (3)	O22^i^—K9—O24	165.65 (3)
O17^xiii^—K3—O2	86.06 (3)	O3—K9—O24	105.39 (3)
O22^i^—K3—O2	96.21 (3)	O22^i^—K9—O16	101.07 (4)
O11^iii^—K3—O2	138.28 (3)	O3—K9—O16	169.42 (3)
O17^xiii^—K3—O21^i^	88.37 (3)	O24—K9—O16	64.73 (3)
O22^i^—K3—O21^i^	54.13 (3)	O22^i^—K9—O15^xiv^	104.22 (3)
O11^iii^—K3—O21^i^	137.29 (3)	O3—K9—O15^xiv^	75.10 (3)
O2—K3—O21^i^	80.11 (3)	O24—K9—O15^xiv^	77.10 (3)
O17^xiii^—K3—O3	124.84 (3)	O16—K9—O15^xiv^	98.19 (3)
O22^i^—K3—O3	84.42 (4)	O22^i^—K9—O12^i^	112.94 (3)
O11^iii^—K3—O3	89.56 (3)	O3—K9—O12^i^	87.10 (3)
O2—K3—O3	52.20 (3)	O24—K9—O12^i^	71.65 (3)
O21^i^—K3—O3	113.19 (3)	O16—K9—O12^i^	92.90 (3)
O19^iv^—K4—O5^iii^	95.09 (3)	O15^xiv^—K9—O12^i^	138.23 (3)
O19^iv^—K4—O6	85.91 (3)	O22^i^—K9—O23^i^	48.17 (2)
O5^iii^—K4—O6	175.02 (3)	O3—K9—O23^i^	101.27 (3)
O19^iv^—K4—O19^xiii^	80.50 (3)	O24—K9—O23^i^	129.23 (3)
O5^iii^—K4—O19^xiii^	90.82 (4)	O16—K9—O23^i^	88.46 (3)
O6—K4—O19^xiii^	94.15 (4)	O15^xiv^—K9—O23^i^	152.38 (2)
O19^iv^—K4—O1^vii^	86.01 (3)	O12^i^—K9—O23^i^	67.43 (2)
O5^iii^—K4—O1^vii^	79.67 (4)		

Symmetry codes: (i) *x*, −*y*+1/2, *z*−1/2; (ii) −*x*, −*y*, −*z*; (iii) *x*, −*y*+3/2, *z*−1/2; (iv) −*x*+1, −*y*+1, −*z*+1; (v) −*x*+1, *y*−1/2, −*z*+1/2; (vi) *x*, −*y*+1/2, *z*+1/2; (vii) −*x*+1, *y*+1/2, −*z*+1/2; (viii) *x*, −*y*+3/2, *z*+1/2; (ix) −*x*, *y*−1/2, −*z*+1/2; (x) −*x*, −*y*+1, −*z*; (xi) *x*, *y*−1, *z*; (xii) *x*, *y*, *z*+1; (xiii) *x*, *y*, *z*−1; (xiv) −*x*, *y*+1/2, −*z*+1/2; (xv) *x*, *y*+1, *z*.

### Table 1 Characterization of K—O coordination spheres; coordination number as well as minimal and maximal distances (Å) within the coordination spheres for each K atom are given.

**Table:**

atom K	coord. number	K—O distances (Å)
K1	8	2.6202 (10)–3.2026 (12)
K2	7	2.5994 (9)–2.9721 (10)
K3	6	2.6337 (11)–2.9790 (11)
K4	6	2.7005 (10)–3.0451 (10)
K5	8	2.6787 (9)–3.3087 (12)
K6	7	2.6690 (10)–3.0404 (9)
K7	7	2.6369 (9)–3.0973 (10)
K8	7	2.5736 (9)–3.0747 (10)
K9	7	2.6965 (9)–3.3094 (11)

### Table 2 Characterization of K—O coordination spheres; the minimal and maximal K—O distances (Å) for the terminal and bridging oxygens (there is only one bridging oxygen in the coordination spheres of K2, K3, and K8).

**Table:**

atom K	K—O_terminal_ / K—O_bridge_ distances (Å)
K1	2.6202 (12)–3.2027 (18) / 3.0287 (14)–3.1684 (16)
K2	2.5994 (12)–2.9722 (14) / 2.8588 (14)
K3	2.6337 (13)–2.9790 (14) / 2.8348 (13)
K4	2.7006 (13)–2.7762 (15) / 2.9576 (15)–3.0451 (13)
K5	2.6787 (11)–3.3088 (19) / 3.1012 (15)–3.2476 (15)
K6	2.6690 (14)–2.9869 (16) / 2.8236 (13)–3.0405 (13)
K7	2.6368 (12)–2.9005 (15) / 2.7891 (14)–3.0974 (13)
K8	2.5737 (12)–3.0747 (13) / 2.9491 (13)
K9	2.6965 (2)–2.8865 (13) / 2.9783 (12)–3.3095 (16)
